# *pax1-1 *partially suppresses gain-of-function mutations in *Arabidopsis AXR3/IAA17*

**DOI:** 10.1186/1471-2229-7-20

**Published:** 2007-04-12

**Authors:** Mimi Tanimoto, Jemma Jowett, Petra Stirnberg, Dean Rouse, Ottoline Leyser

**Affiliations:** 1 Department of Biology, University of York, Heslington, York, YO10 5YW, UK; 2 Department of Molecular and Cellular Biology, Axelrod Building, University of Guelph, Guelph, Ontario, N1G 2W1, Canada; 3 Section of Molecular and Cellular Biology, University of California, Davis, One Shields Avenue, Davis, CA 95616, USA; 4 Research School of Biological Science, GPO Box 475, Canberra, ACT 2601, Australia

## Abstract

**Background:**

The plant hormone auxin exerts many of its effects on growth and development by controlling transcription of downstream genes. The *Arabidopsis *gene *AXR3/IAA17 *encodes a member of the Aux/IAA family of auxin responsive transcriptional repressors. Semi-dominant mutations in *AXR3 *result in an increased amplitude of auxin responses due to hyperstabilisation of the encoded protein. The aim of this study was to identify novel genes involved in auxin signal transduction by screening for second site mutations that modify the *axr3-1 *gain-of-function phenotype.

**Results:**

We present the isolation of the *partial suppressor of axr3-1 *(*pax1-1*) mutant, which partially suppresses almost every aspect of the *axr3-1 *phenotype, and that of the weaker *axr3-3 *allele. axr3-1 protein turnover does not appear to be altered by *pax1-1*. However, expression of an *AXR3*::*GUS *reporter is reduced in a *pax1-1 *background, suggesting that *PAX1 *positively regulates *AXR3 *transcription. The *pax1-1 *mutation also affects the phenotypes conferred by stabilising mutations in other Aux/IAA proteins; however, the interactions are more complex than with *axr3-1*.

**Conclusion:**

We propose that *PAX1 *influences auxin response *via *its effects on *AXR3 *expression and that it regulates other *Aux/IAA*s secondarily.

## Background

The phytohormone auxin is central to the regulation of plant growth and development. Processes controlled by auxin include apical dominance, adventitious root formation, tropic responses, vascular patterning and root hair development [1-6]. These diverse morphological events are brought about by changes in cell division, expansion and differentiation [7-9], and many of them are mediated by the ability of auxin to control gene expression. Several families of genes containing Auxin Response Elements (AuxREs) in their promoters, are rapidly transcriptionally upregulated as a primary response to auxin [10], including members of the *Aux/IAA *gene family.

The *Aux/IAA *genes form a large multi-gene family found throughout the plant kingdom [11-14]. There are 29 members in *Arabidopsis *and their expression varies with respect to tissue specificity, auxin induction kinetics and sensitivity to auxin dose [11,15]. Aux/IAA proteins are characterised by four highly conserved domains [11]. C-terminal domains III and IV mediate homo- and hetero-dimerisation between Aux/IAA proteins [16]. Domains III and IV are also found in the Auxin Response Factor (ARF) family of transcription factors, a subset of which activate transcription from AuxRE-containing promoters [17,18]. Conservation of domains III and IV between the Aux/IAAs and ARFs allows combinatorial dimerisation amongst these families. The N-terminal domain I of Aux/IAA proteins functions as a transcriptional repression domain [19]. Thus, dimers between Aux/IAAs and activator ARFs block gene transcription [19-22].

Most Aux/IAA proteins are extremely unstable, with half-lives ranging from 6 to 80 minutes [23-26]. They interact *via *domain II with SCF^TIR1 ^(Skp1, Cdc53/cullin, F-box protein^TIR1^) and other auxin receptor F-box-containing E3 ubiquitin-protein ligase complexes, which target them for degradation by the ubiquitin-proteasome pathway [25,27]. Auxin promotes their association with such E3s, increasing their turnover [25,27-29]. Thus transcriptional auxin responses are mediated by the auxin-induced destabilisation of Aux/IAA proteins, which relieves associated activator ARFs from repression and in turn, upregulates transcription from AuxREs [30]. Since *Aux/IAA *genes contain AuxREs in their promoters, this provides a feedback mechanism for regulating their own expression. Furthermore, it allows *Aux/IAA*s to control each other's expression creating a complex network of cross regulation amongst the family.

Dominant or semi-dominant mutations resulting in hyper-stabilisation of individual Aux/IAAs have been isolated in at least 8 *Aux/IAA *genes from *Arabidopsis *[31-38]. Such stabilising mutations occur in domain II and act by reducing the affinity of the Aux/IAA for SCF^TIR1^, conferring pleiotropic auxin related phenotypes. For *AXR3/IAA17*, two such gain-of-function alleles have been described [39]. *axr3-1 *and *axr3-3 *plants show increased apical dominance, increased adventitious root formation, agravitropic roots, no root hairs, and epinastic petioles. These phenotypes are generally consistent with an increase in the magnitude of auxin responses. However, similar mutations in other *Aux/IAA *genes cause reduced auxin responses in some tissues and increased responses in other tissue types [31-38].

Although the study of the *Aux/IAA *and *ARF *families has elucidated many of the early events in auxin signal transduction, there is an ongoing requirement to find upstream regulators and novel targets of Aux/IAA and ARF regulated transcription. Here we present the isolation and characterisation of an extragenic recessive mutant, *pax1-1*, which partially suppresses the phenotypes of semi-dominant *axr3 *alleles. The *pax1-1 *single mutant shows pleiotropic phenotypes consistent with altered auxin responses. Double mutant analyses suggest that *PAX1 *also interacts genetically with other members of the *Aux/IAA *family. We propose that *PAX1 *acts upstream of *Aux/IAA *genes by regulating their transcription.

## Results

### Mutant isolation

**Figure 1:**
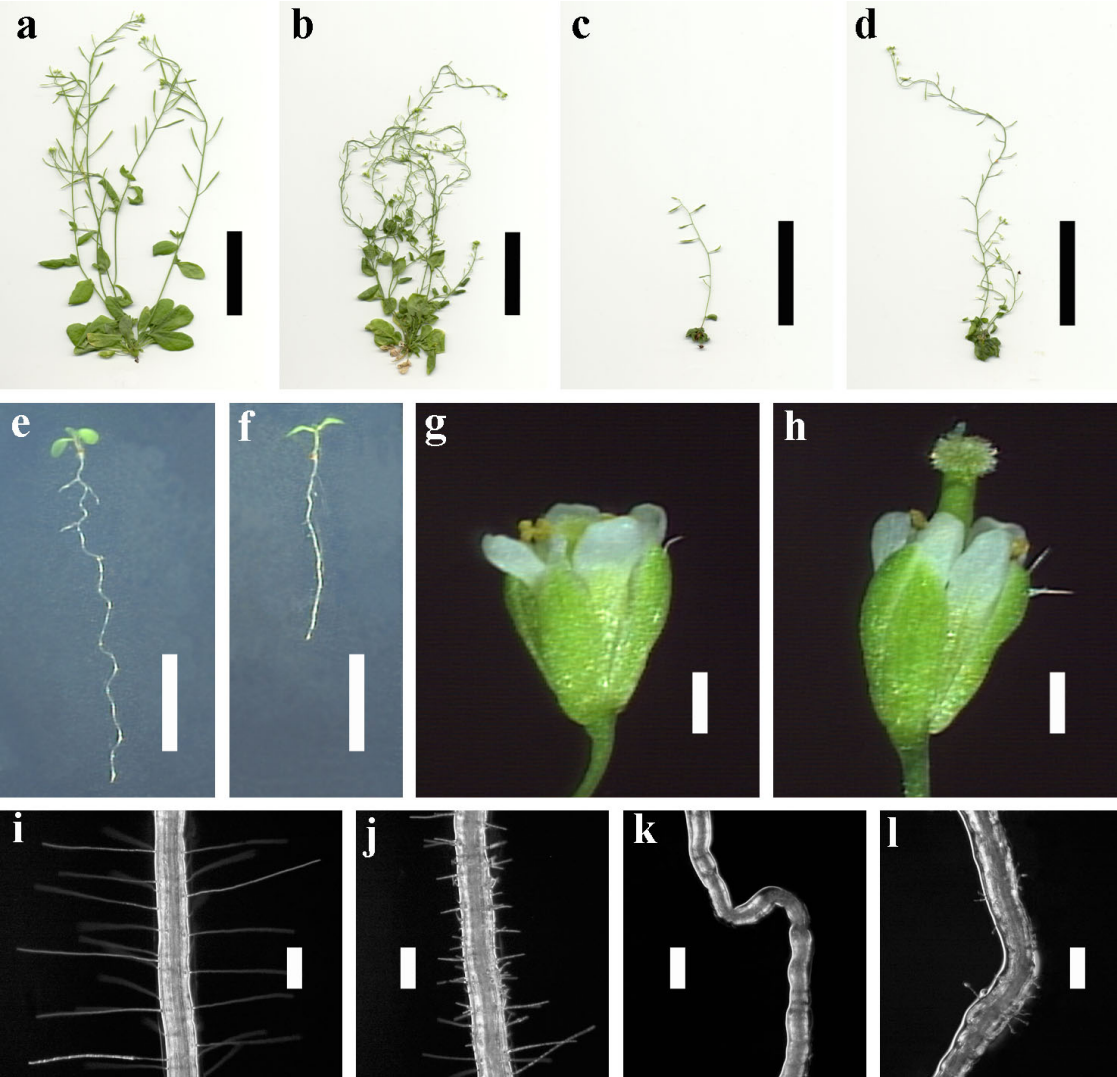
***pax1-1 *mutant phenotypes in wild-type and *axr3 *backgrounds**. (a) Wild type, (b) *pax1-1*, (c) *axr3-1 *and (d) *axr3-1 pax1-1 *shoot morphology at maturity. Bar = 5 cm. (e) Wild type and (f) *pax1-1 *seedling 6 days after germination (dag). Bar = 1 cm. (g) Wild type and (h) *pax1-1 *flower. The elongated carpel phenotype seen in *pax1-1 *is also observed in *axr3 pax1-1 *double mutants. Bar = 0.5 mm. (i) Wild type, (j) *pax1-1*, (k) *axr3-1 *and (l) *axr3-1 pax1-1 *root hairs 3 dag. In *pax1-1 *and *axr3-1 pax1-1*, multiple roots hairs arise from a single root epidermal cell. Bar = 250 μm. All *axr3-3 pax1-1 *phenotypes were qualitatively similar to *axr3-1 pax1-1*, showing suppression of *axr3 *phenotypes by *pax1-1*.

To search for new mutants altered in auxin signalling and more specifically, to find novel genetic interactors with *AXR3*, we screened x-ray-mutagenised *axr3-1 *(Columbia) plants for reversion of their strict apical dominance. The mutagenised seed were also homozygous for the *gl1-1 *mutation (which causes plants to lack leaf trichomes) so that revertant lines could be distinguished from contaminating wild-type plants. One line recovered from the screen showed increased numbers of axillary branches arising from the shoot, compared with *axr3-1 *controls (Figure 1c and 1d). The dwarf phenotype and dark green, curled leaves caused by the *axr3-1 *mutation were also partially suppressed in the revertant. In addition, the carpels were relatively longer than the sepals and petals, such that they protruded from the closed flower buds. Revertant plants were outcrossed to wild-type (Columbia) plants and segregation of individuals in the F_2 _generation morphologically similar to *axr3-1 *homozygotes indicated that the suppressor mutation is extragenic to *axr3*. Thus, the new mutant was named *partial suppressor of axr3-1 (pax1-1*). Segregation analysis also suggested that the *pax1-1 *mutation is recessive to wild type and that it is linked to *axr3-1 *(data not shown).

### Morphological phenotypes of *pax1-1 *seedlings

**Figure 2:**
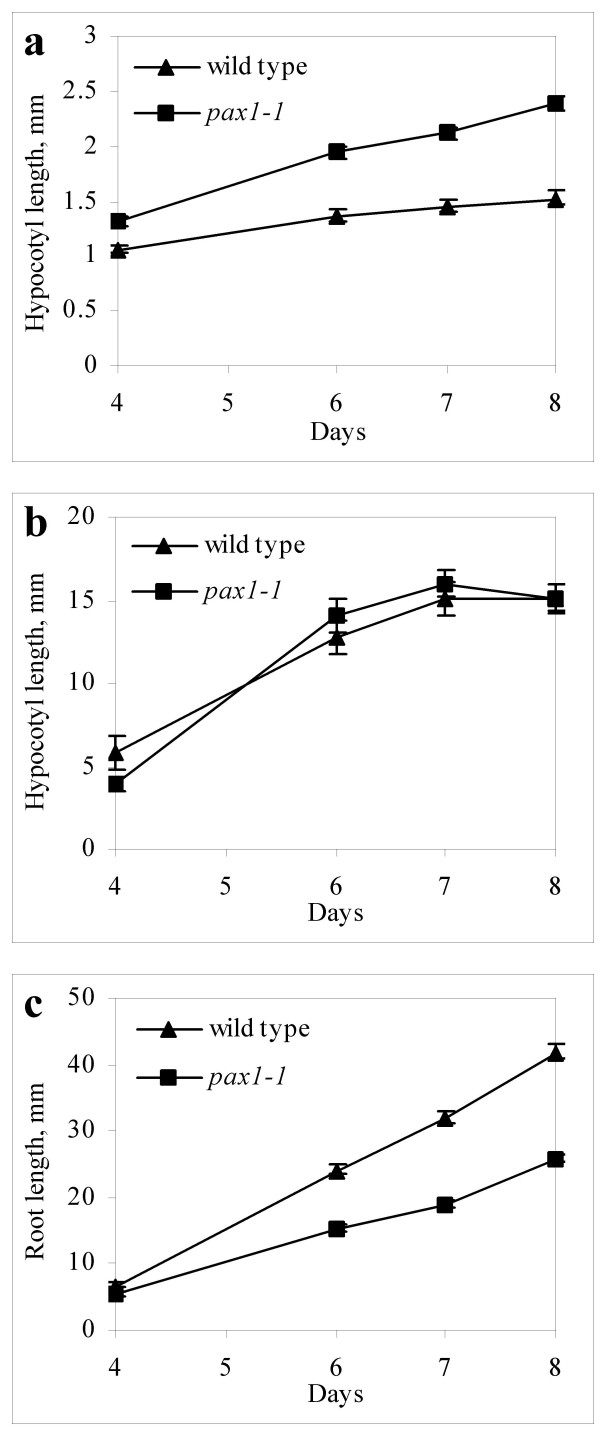
***pax1-1*seedling growth rates**. (a) Hypocotyl elongation in light grown seedlings. n ≥ 15. (b) Hypocotyl elongation in etiolated seedlings. n ≥ 9. (c) Root elongation in light grown plants. n ≥ 19. Measurements were taken at various times after sowing. Error bars represent standard errors of the means (SE).

To gain insight into the wild-type role of *PAX1*, a detailed analysis of the *pax1-1 *mutant phenotype in a wild-type genetic background was carried out. Germination is slower in *pax1-1 *mutants compared with wild-type seeds (data not shown). *pax1-1 *hypocotyls are longer than wild-type when grown under a long day photoperiod, as a result of an increased growth rate (Figure 1e and 1f, Figure 2a). In contrast, dark grown hypocotyl elongation was not significantly different from wild-type (Figure 2b). However, the rate of root growth is reduced in *pax1-1 *mutants (Figure 2c), and *pax1-1 *roots are abnormally straight, indicating that they have a reduced root wave response (Figure 1e and 1f).

*pax1-1 *plants produce root hairs at a higher density than wild-type and with altered morphology (Figure 1i and 1j). *pax1-1 *hairs are approximately 34% shorter than wild-type root hairs (wild type = 379 ± 1.16 μm, *pax1-1 *= 252 ± 0.96 μm), and develop in aberrant patterns. In wild-type *Arabidopsis*, the root epidermis is arranged in longitudinal files of cells [40]. Each file is composed entirely of either hair-forming cells (trichoblasts) or non-hair-forming cells (atrichoblasts), and each trichoblast forms a single root hair. However, in *pax1-1 *some trichoblasts produce multiple hairs, with up to 5 hairs observed per cell (Fig 1j). These hairs arise from a single initiation site. Thus, the increase in root hair density can be attributed at least partially to the development of multiple hairs from some trichoblasts. Also, some hairs form branches during the rapid tip growth phase of hair elongation, resulting in stalked, branched structures. Therefore *PAX1 *appears to affect both the orientation and the amount of root hair elongation.

### Auxin response of *pax1-1 *mutants

**Figure 3:**
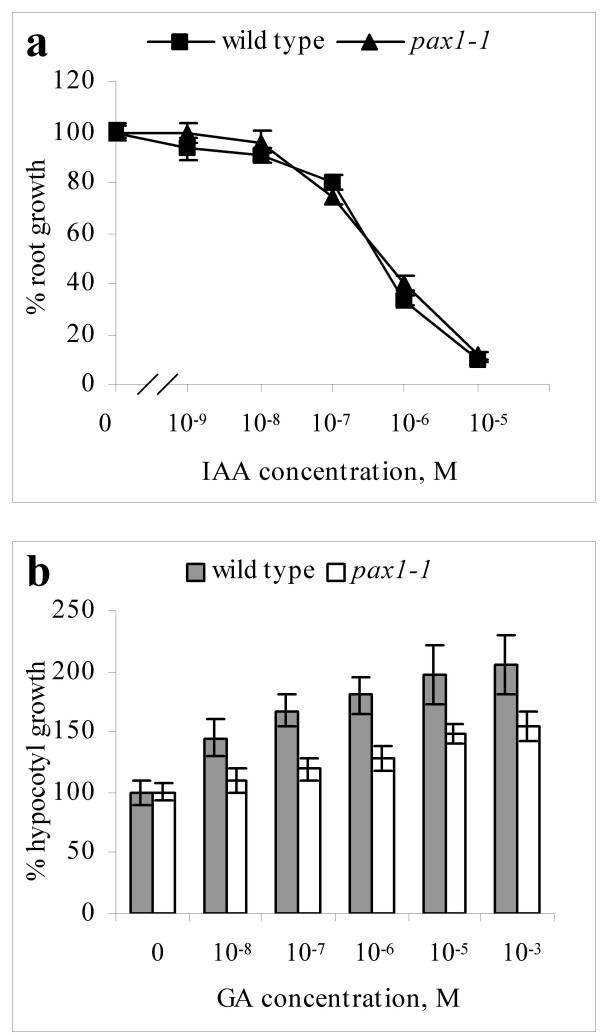
***pax1-1 *hormone responses**. (a) Root growth response to exogenous IAA. n ≥ 13. (b) Hypocotyl growth response to exogenous GA_3_. n ≥ 14. The amount of growth is expressed as percentage growth relative to untreated controls. Error bars represent SE.

To test the auxin response of *pax1-1 *roots, seedlings were transferred to medium containing various concentrations of the natural auxin, indole-3-acetic acid (IAA) and root elongation was measured 5 days later. On increasing concentrations of IAA between 10 nM and 10 μM, wild-type root growth was inhibited in a dose-dependent manner (Figure 3a). *pax1-1 *root elongation follows a similar dose response to IAA as wild-type.

### *PAX1 *and *AXR3 *interact genetically

**Figure 4:**
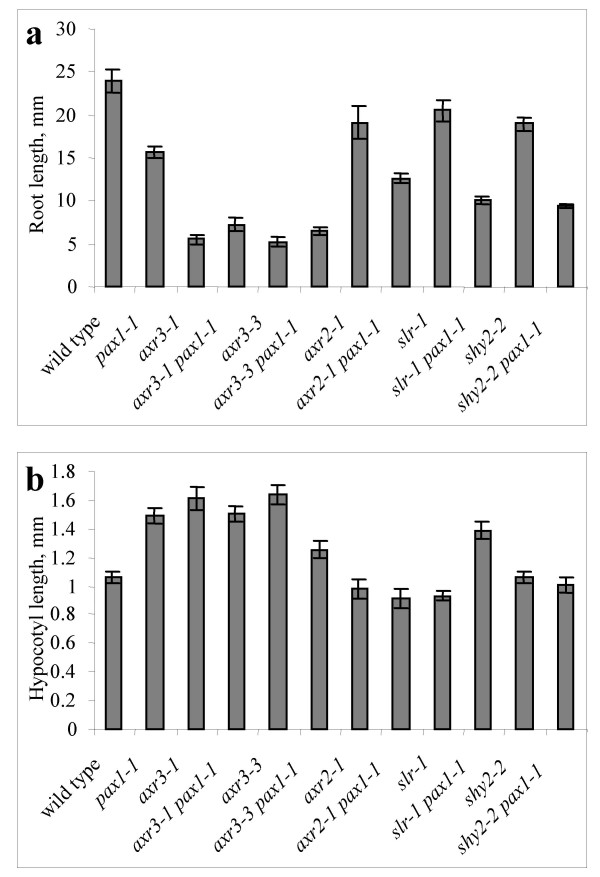
**Double mutant analysis**. (a) Root length. n ≥ 10. (b) Hypocotyl length. n ≥ 8. Measurements were taken 5 dag. Error bars represent SE.

To characterise in more detail the effect of *pax1-1 *on the *axr3-1 *phenotype, we re-crossed *pax1-1 *into an *axr3-1 *background. We also constructed double mutants between *pax1-1 *and a second, weaker semi-dominant allele, *axr3-3*, in order to assess the allele specificity of suppression. *axr3-1 *and *axr3-3 *show severely reduced root growth compared with wild-type (Figure 4a) [39]. However, in double mutants with *pax1-1*, root elongation is increased by 32 percent and 23 percent, respectively. This effect is particularly striking considering that the *pax1-1 *mutation causes a reduction in root length when in a wild-type background. Thus, *PAX1 *appears to be required for the full inhibition of root growth by *axr3 *gain-of-function alleles. Other aspects of the root phenotype are also suppressed by *pax1-1*. In contrast to the *axr3 *single mutants, where root growth apparently wanders randomly with frequent changes in direction (e.g. kink in root in Figure 1k), the *axr3-1 pax1-1 *and *axr3-3 pax1-1 *double mutant roots grow more towards the gravity vector, and produce more root hairs (Figure 1k and 1l).

With respect to hypocotyl elongation, a similar interaction to that observed with root growth occurs. During the first few days of growth *axr3-1 *hypocotyls elongate at an increased rate compared with wild-type, followed by a period of slower elongation [41]. Five days after germination, *axr3-1 *and *axr3-3 *hypocotyls are slightly longer than wild-type, similar to *pax1-1 *(Figure 4b). However, *axr3-1 pax1-1 *hypocotyls are no longer than either of the single mutants and *axr3-3 pax1-1 *hypocotyls are shorter than either single mutant.

**Figure 5:**
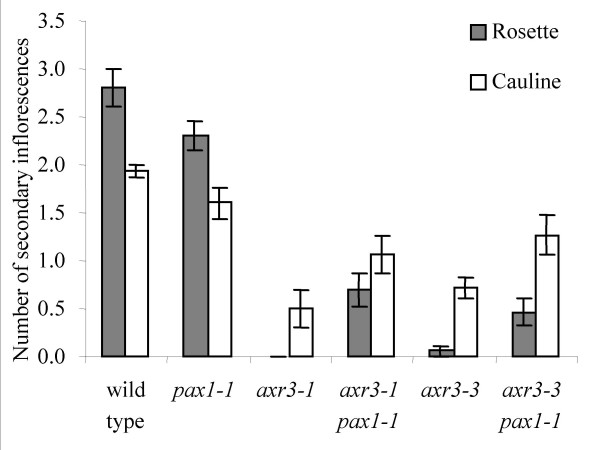
**Shoot branching phenotypes of *axr3 pax1-1 *double mutants**. Numbers of secondary inflorescences arising from rosette and cauline nodes following flowering. n ≥ 8. Error bars represent SE.

All of the *axr3 *shoot phenotypes analysed were partially suppressed by *pax1-1*. As in the original *axr3-1 pax1-1 *line isolated from the screen, *pax1-1 *suppresses the increase in apical dominance caused by *axr3-1 *(Figure 1c and 1d, Figure 5). *axr3-1 pax1-1 *double mutants produce more secondary inflorescences from rosette and cauline nodes compared with *axr3-1 *homozygotes. Conversely, *pax1-1 *single mutants have slightly fewer second order inflorescences than wild-type plants. Therefore, *pax1-1 *has opposite effects on shoot branching in *axr3-1 *and wild-type backgrounds. Similar results were obtained for the *axr3-3 pax1-1 *double mutant (Figure 5). *axr3-1 pax1-1 *and *axr3-3 pax1-1 *plants are less dwarfed than *axr3-1 *and *axr3-3*, respectively, despite the fact that *pax1-1 *causes a dwarf phenotype in plants that are wild-type for *AXR3 *(Figure 1a to 1d). Also, the leaves of each double mutant are paler green, less epinastic and less curled than the *axr3 *parent (Figure 1c and 1d).

In summary, *pax1-1 *partially suppresses almost every *axr3-1 *and *axr3-3 *phenotype.

### Mechanism of suppression

**Figure 6:**
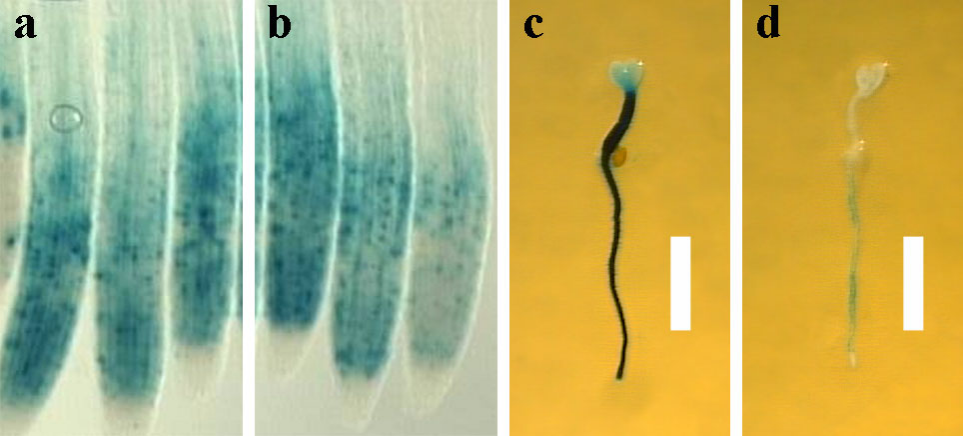
**Control of *AXR3* expression by *PAX1***. HS::axr3-1NT-GUS expression in (a) wild type and (b) *pax1-1*, 2 h following heat induction. *AXR3*::GUS expression in (c) wild type and (d) *pax1-1 *seedlings stained for GUS activity 2 dag. Bar = 2 mm.

Two possible mechanisms by which *pax1-1 *may suppress the *axr3-1 *phenotype are: (i) by increasing the degradation rate of the over-stable axr3-1 protein, or (ii) by decreasing the level of *axr3-1 *transcription. To test the first possibility, *pax1-1 *was crossed with transgenic plants expressing amino-terminal domains I and II of axr3-1 (axr3-1NT) fused to the β-glucuronidase (GUS) reporter, under the control of the soybean heat shock promoter (HS) [25]. This construct is an established reporter for axr3-1 protein stability. Plants were heat shocked at 37°C for 2 h and stained for GUS activity at various intervals following heat shock, to monitor the rate of GUS turnover. In wild-type plants GUS activity remained constant between 30 min and 2 h following heat shock, showing that the axr3-1 protein is not observably degraded during this time period (Figure 6a). Similar results were observed in a *pax1-1 *background suggesting that *pax1-1 *has no effect on axr3-1 turnover (Figure 6b). In another experiment, no difference was observed between wild type and *pax1-1 *on the turnover of AXR3NT-GUS, in which the wild-type domain II destabilisation sequence is present (data not shown) [25].

To assess whether *pax1-1 *controls *AXR3 *transcription, *pax1-1 *was crossed to transgenic plants containing a transcriptional fusion between the *AXR3 *promoter and the *GUS *reporter gene (*AXR3::GUS*). Seedlings were stained for GUS activity two days after germination. In a wild-type background, GUS activity was observed in the root and hypocotyl (Figure 6c). However, *AXR3::GUS *expression was significantly reduced in *pax1-1 *(Figure 6d). GUS staining was almost completely absent from the hypocotyl and was greatly reduced in the root.

### *PAX1 *interacts genetically with *Aux/IAA *genes

To determine whether *PAX1 *interacts with other *Aux/IAA *genes, we created double mutants between *pax1-1 *and *axr2-1*, *slr-1 *and *shy2-2 *[31,42,43]. These mutants carry semi-dominant mutations in domain II of *IAA7*, *IAA14 *and *IAA3*, respectively [31,33,37]. Like *axr3-1*, the mutations result in gain-of-function phenotypes due to hyperstabilisation of the encoded proteins. Analysis of the double mutant phenotypes showed effects that could be classified into two different categories (Figure 4).

In the first class, the two mutations produced an additive effect. For example, the root length of the *axr2-1 pax1-1 *double mutant is less than that of both the *axr2-1 *and *pax1-1 *single mutants, which in turn are both less than wild-type (Figure 4a). This suggests that *AXR2/IAA7 *and *PAX1 *function independently in root elongation, although since the *axr2-1 *mutation is a dominant gain-of-function allele these results must be interpreted with caution. Likewise, *slr-1 *and *pax1-1 *had an additive effect on both root and hypocotyl elongation, implying that the two genes act independently during these processes (Figure 4a and 4b). The effects of *shy2-2 *and *pax1-1 *on root elongation were also additive.

In the second class of genetic interaction, one of the mutations was epistatic to the other. For example, under our growth conditions, five days after germination *axr2-1 *and *axr2-1 pax1-1 *hypocotyls were approximately the same length, with *axr2-1 *suppressing the long hypocotyl phenotype of *pax1-1 *(Figure 4b). *shy2-2 *was also epistatic to *pax1-1 *with respect to hypocotyl elongation.

### *PAX1 *and gibberellic acid response

Several aerial features of the *pax1-1 *phenotype are suggestive of defects in gibberellic acid (GA) response. These include slow germination, short stature, dark green leaves (Figure 1a and 1b), protruding pistils (Figure 1g and 1h) and features indicative of slower phase change [44-47]. Plants pass through several distinct phases of development, marked by morphological changes in the lateral organs produced by the shoot apical meristem. In *Arabidopsis*, the first two rosette leaves are relatively round and each subsequent leaf becomes progressively more elongated [48]. *pax1-1 *produces rounder leaves than wild type, suggesting that it remains for longer in more juvenile phases of development. To test this, other markers of phase change were compared in *pax1-1 *and wild-type plants. Juvenile leaves form trichomes on their adaxial (dorsal) surfaces but not their abaxial (ventral) surfaces, and the transition from the juvenile to the adult phase of vegetative development is marked by the onset of trichome production on the abaxial surfaces of the leaves [46]. Under our growth conditions, wild-type plants produced 5.80 ± 0.09 juvenile leaves, lacking abaxial trichomes, whereas *pax1-1 *plants produced 8.60 ± 0.37 (Table 1). As the rate of *pax1-1 *leaf initiation is reduced, this suggests that the transition to the adult growth phase is delayed both developmentally and temporally. Furthermore, floral transition also occurs both developmentally and temporally later than wild type, although this difference is mostly due to the longer juvenile phase of *pax1-1 *(Table 1).

To test more directly for defects in GA response, *pax1-1 *plants were assayed for hypocotyl elongation on medium supplemented with GA_3_. Wild-type hypocotyl elongation was stimulated in a dose-dependent manner by exogenous GA_3 _(Figure 3b). However in *pax1-1*, GA-induced growth is reduced compared with wild type.

### Genetic mapping

From the F_2 _of the outcross of the original *pax1-1 axr3-1 *double mutant to wild type (Columbia) *PAX1 *was estimated to map approximately 20 cM proximal to *AXR3 *on chromosome 1. To refine the map position of the *PAX1 *locus, *pax1-1 *(Columbia) plants were crossed to plants of the Landsberg *erecta *(L*er*) ecotype and the F_2 _generation was scored for segregation of polymorphic molecular markers between the two ecotypes. Segregation analysis revealed that *PAX1 *maps to a 420 kb region between markers SNP82 (3 recombinants/964 chromosomes) and cer474010 (3 recombinants/964 chromosomes). The region appears to be somewhat recombinationally suppressed, with an estimated 677 kb per cM. Attempts to identify the gene by transformation rescue were thwarted by the discovery that the mutant phenotype is unstable, reverting to wild type at a low frequency that was substantially enhanced by the transformation process (data not shown).

The delimited region includes 113 genes, of which only *ARF19 *is a clear *PAX1 *candidate, given its predicted role in the regulation of *Aux/IAA *gene expression. However, DNA sequencing of the *ARF19 *locus, including 1.5 kb upstream of the predicted translation start, from *pax1-1*, revealed no mutations. Furthermore *arf19 *insertion mutants were reported to have no phenotype [49,50], and trans-heterozygotes between an *arf19 *mutant and *pax1-1 *are phenotypically wild-type (data not shown) suggesting that *PAX1 *and *ARF19 *are not allelic. Thus, *PAX1 *is likely to be a previously unknown component of the *Aux/IAA *regulatory network.

## Discussion

### *PAX *suppresses *axr3 *gain-of-function alleles

In this paper we present the isolation and characterisation of a new *Arabidopsis *mutant, *pax1-1*, which partially suppresses the phenotype of *axr3 *gain-of-function alleles. Virtually every aspect of the *axr3 *mutant phenotype analysed is suppressed at least partially by *pax1-1*. This is particularly compelling in cases where *pax1-1 *confers a more wild-type phenotype on *axr3 *mutants, despite having the opposite effect in a wild-type background. Examples of such are during root and hypocotyl elongation, and in the outgrowth of axillary inflorescences. These results suggest that *PAX1 *may encode a general positive regulator of *AXR3 *action. *PAX1 *does not appear to act at the level of AXR3 protein stability since axr3-1NT-GUS and AXR3NT-GUS translational fusions were turned over at similar rates in *pax1-1 *compared to wild type. In contrast, expression of an *AXR3::GUS *reporter was down-regulated in a *pax1-1 *mutant background suggesting that in wild-type plants, *PAX1 *regulates *AXR3 *transcription positively. Consistent with this idea, suppression of the *axr3 *phenotypes is not allele specific, as might be expected if the effect was at the protein level.

This suggests a model for the suppression of *axr3-1 *and *axr3-3 *phenotypes by *pax1-1*. Such phenotypes are caused by AXR3 hyperstabilisation leading to the accumulation of increased protein levels. Therefore, reduced *AXR3 *transcription in *pax1-1 *would result in lower levels of AXR3 protein accumulation, and thus weaker *axr3 *phenotypes. Although this model is attractive, we have been unable to detect reliable differences from wild-type in the steady state levels the endogenous *AXR3 *mRNA in the *pax1-1 *mutant background (JJ and HMOL unpublished results). This might be because of the differences are tissue specific and therefore less easy to detect by RT-PCR than by histochemical GUS staining. However, it is also possible that the *AXR3::GUS *reporter does not accurately reflect the expression of the endogenous gene, as is often the case with promoter-GUS reporters. Whilst this would argue against a model of *axr3-1 *suppression by specific transcriptional down-regulation of *AXR3*, our results none the less suggest that the *pax1-1 *phenotype may be mediated by changes in transcription of auxin-regulated genes, since the reporter construct is rapidly auxin responsive (MT and HMOL unpublished results).

### *PAX1 *and other *Aux/IAAs*

Double mutant analysis demonstrates that *PAX1 *interacts genetically with other members of the *Aux/IAA *family in addition to *AXR3*. However, unlike its interaction with *AXR3*, where virtually all phenotypes are suppressed, a more complex set of interactions is observed with the other *Aux/IAA*s tested.* pax1-1 *shows combinations of epistatic and additive phenotypes with *axr2-1*, *slr-1 *or *shy2-2*. Furthermore, the type of interaction *pax1-1 *has with different *Aux/IAA *mutants, varies in different organs.

One explanation for the more complex set of interactions observed is that the effects of *PAX1 *on other members of the *Aux/IAA *family are indirect. Since *Aux/IAA *genes regulate each others' transcription, alterations in the expression level of one *Aux/IAA *gene (i.e. *AXR3*) could have downstream and feedback effects on the transcription of other family members. Therefore, the primary targets of *PAX1 *may include *AXR3 *transcription, whereas the effect on other *Aux/IAA*s may be secondary. Thus in a *pax1-1 *background, the phenotypes may result from widespread alterations in the balance of the Aux/IAA-ARF network, triggered by primary changes in just a few genes.

### *pax1-1 *is defective in auxin-regulated development

A prediction of our model is that *pax1-1 *should have auxin-related phenotypes in a wild-type background. Indeed, many aspects of the *pax1-1 *phenotype are reminiscent of defects in auxin transport or signal transduction. For example, root, hypocotyl and stem elongation are regulated by auxin, and mutations in components of auxin signalling, such as *Aux/IAA *and *ARF *family members, lead to perturbations in these processes [39,51,52]. A mutant in *ARF2 *flowers late, suggesting that auxin may also control floral transition [53]. Another auxin-mediated process is root waving, with transport and signalling mutants displaying abnormal patterns of waving [37,54]. Furthermore, exogenous auxin affects root hair elongation and morphology, whilst many auxin response mutants show altered root hair growth [3,55,56].

Although the data discussed above suggest that *PAX1 *is involved in auxin response, *pax1-1 *roots show a wild-type growth response to exogenous IAA. This is consistent with the idea that the Aux/IAA-ARF network is differently configured but not globally down-regulated in the mutant, so that some phenotypes are suggestive of auxin resistance, but others are not. Analysis of the loss of function phenotypes of individual *Aux/IAA*s and *ARF*s demonstrates that the network is very robust with significant functional redundancy. If the *PAX1 *gene acts to modulate the network, it is therefore likely to affect more than one network member.

### *PAX1 *and GA response

Another aspect of the *pax1-1 *phenotype is apparent defects in GA response. GA promotes germination and phase change, and increases stem and floral organ elongation [44-47]. *pax1-1 *plants show decreased germination, delayed phase change, reduced stem length, and altered floral organ elongation. Consistent with *PAX1 *functioning in GA responses, mutant hypocotyls treated with GA are resistant to its growth-promoting effects.

These data implicate *PAX1 *in both auxin and GA responses. Auxin is required for GA signalling, by regulating the GA-induced degradation of DELLA growth repressor proteins [57]. Furthermore, GA mediated destabilisation of the DELLA protein RGA is reduced in the auxin resistant mutant, *axr1-12*. *AXR1 *encodes a regulator of an E3 ubiquitin-protein ligase responsible for Aux/IAA turnover, and thus AXR1 may control DELLA protein levels through the destabilisation of Aux/IAAs [25,58]. In such a case, effects of *PAX1 *on *Aux/IAA *expression levels could be sufficient to alter DELLA protein turnover. In addition, GA metabolism may be affected since AXR3 and other Aux/IAAs are thought to regulate transcription of GA metabolism genes directly[59]. Alternatively, *PAX1 *might control GA signalling and/or metabolism independently of its effects on Aux/IAA protein levels.

## Conclusion

Genetic analysis of the *pax-1-1 *mutant demonstrates that *PAX1 *positively regulates *AXR3/IAA17 *transcription. *PAX1 *also interacts genetically with other *Aux/IAA*s, although these effects may occur secondarily through its ability to regulate *AXR3*. In addition, GA responses are clearly affected by *PAX1*. Thus the *PAX1 *locus is important for both auxin and GA signalling. Understanding the mechanisms that underlie cross talk between different plant hormones is currently of great interest to plant biologists. Thus the cloning and molecular analysis of *PAX1 *should have valuable implications for the hormone signalling community.

## Methods

### Plant materials and growth conditions

The following mutants are in the Columbia (Col) ecotype: *axr3-1*, *axr3-3*, *axr2-1*, and *slr-1 *[31,39,43]. *shy2-2 *is in the L*er *ecotype [42]. Wild-type L*er *was used for mapping experiments.

Seeds were sterilised and sown onto Petri dishes containing agar-solidified *Arabidopsis thaliana *salts (ATS) growth medium [43]. For examination of root hair phenotypes, the agar was replaced with 3.6% Phytagel (Sigma, UK). Plants were grown at 17–25°C in white light (60–90 μmol m^-2^s^-1^), under a 16 h light/8 h dark photoperiod. Seedlings were transplanted to Levington F2 compost (Fisons, UK) 8–10 days after germination, and grown to maturity under the conditions described above.

### Mutant isolation

An M_1 _population of 50 000 *axr3-1 gl1-1 *plants was generated by x-ray mutagenesis of the seed (8 kR dose). The M_2 _was harvested as 30 seed pools, and 1500 plants from each pool were screened for suppression of the *axr3-1 *shoot phenotype. The *pax1-1 *single mutant was obtained from the F_2 _of an outcross between *axr3-1 pax1-1 *plants and wild-type (Col). *pax1-1 *homozygotes were backcrossed twice to wild type and for each backcross, the F_2 _generation was analysed for segregation of the mutant phenotypes. 55 and 94 F_2 _plants were analysed from the first and second backcrosses, respectively, and all of the phenotypes cosegregated. *pax1-1 *homozygotes from the second backcross were used in all experiments except for the genetic crosses and in Figure 3a, where the original *pax1-1 *single mutant line was used.

### Genetic mapping

*pax1-1 *was crossed with L*er *plants and F_2 _individuals showing the *pax1-1 *mutant phenotype were selected to form the mapping population. Genomic DNA was isolated from individual plants and amplified with primers for SSLP, CAPS and SNP markers listed at The *Arabidopsis *Information Resource (TAIR) [60]. Primer sequences and restriction sites for markers nga392, M59 and CAT3 were obtained from TAIR. Primer sequences for the remaining markers were as follows: cer465593: 5' caacaatggtgatatttgttttgc 3' and 5' caacttttaggctctctagcgttt 3'; cer453516: 5' tatcagcaaattgcaaggattaga 3' and 5' tcaccactttgtattgtttttcct 3'; cer465605: 5' tgggagttccaatgtttaaag 3' and 5' attgatggaatggaacagaga 3'; cer452156 5' acacgaccaagaagtcaaata 3' and 5' acaattcttgtcgggcagat 3'; cer474010 5' cgaccctcgagaaagaacaa 3' and 5' gttatactgcgcctggaacc 3'; cer453463: 5' aataaaggcccatcttgtgtgt 3' and 5' actggagcgtcgtcattagttt 3'; cer453259: 5' ggtccaaacaaaaacaaattcc 3' and 5' cgaacaatcaagccacctct 3'; SNP82: 5' tggaaagccattgatggaagg 3' (Col) or 5' tggaaagccattgatggaagc 3' (L*er*), and 5' ttccgaagaccagaataacca 3'; SM106: 5' tatataagagaagagaaaga 3' and 5' gctgagtgagacccagtcct 3', SacI (New England Biolabs) was used to cleave the PCR product.

### Double mutant isolation

To verify the genotypes of double mutants, each line was backcrossed to both *pax1-1 *and wild type. In test crosses to wild type, the phenotype of all F_1 _plants was qualitatively identical to the dominant mutant. F_1 _plants from backcrosses to *pax1-1 *were all morphologically similar to the respective double mutant.

When double mutants between *pax1-1 *(Col) and *shy2-2 *(L*er*) were constructed, double mutants lacking the *erecta *(*er*) mutation were selected for phenotypic analysis. As a control, *shy2-2 *homozygotes lacking the *er *mutation were also selected from the F_2 _of the cross. To verify that these plants were homozygous for the wild-type *ER *allele, they were backcrossed to *shy2-2 *(L*er*) and the F_1 _generation was scored for the absence of the *er *phenotype.

### Phenotypic analysis

In all experiments except those shown in Figure 2, only seedlings germinating within 3 days after sowing were analysed. Root and hypocotyl growth was measured using Lucia G (version 3.52a, 1991, Laboratory Imaging, Nikon UK Limited, Kingston, UK) image analysis software with a video camera input. Timing of floral transition was scored as the number of days after germination (dag) when floral buds first became visible, and the number of leaves at floral transition was scored at least 7 days later. Leaf senescence was recorded every 2 to 3 days so that the number of leaves counted reflected the total number of leaves produced. To characterise shoot branching phenotypes plants were analysed every 2 to 3 days for the time at which the first flower opened, and the numbers of secondary inflorescences were counted 14 days after this. Only axillary buds that were at least 3 mm long were counted as inflorescences.

### Hormone response assays

For the auxin growth response assay, seedlings were germinated on ATS plates and then transferred after 3 days, to new plates supplemented with IAA. The position of each root tip was marked and after a further 5 days, the amount of new root growth was measured.

To test the hypocotyl growth response to GA, seedlings were germinated on ATS plates and then transferred after 4 days to new plates supplemented with GA_3_. The position of each hypocotyl apex was marked and the amount of new hypocotyl growth was measured after a further 4 days.

### Transgenic plants

*Arabidopsis *lines containing the HS::axr3-1NT-GUS and HS::AXR3NT-GUS constructs have been described previously [25].

A genomic fragment containing 2.0 kb 5' to the *AXR3 *translation start was cloned into pBI101.3 (Clontech, UK) upstream of the GUS reporter gene, at *BamHI *and blunted *EcoRI/HindIII *sites, to create the plasmid pAXR3::GUS. This 2.0 kb promoter region was assumed to be sufficient to confer wild-type *AXR3 *expression since transgenic *Arabidopsis *plants containing the *axr3-1 *cDNA fused directly downstream of this promoter displayed phenotypes qualitatively similar to *axr3-1 *mutants. pAXR3::GUS was transformed into wild type (Col) by vacuum infiltration [61]. Multiple independent transgenic lines showed the same qualitative pattern of GUS expression. One of these lines was crossed into *pax1-1*.

### Histochemical localisation of GUS

GUS activity was detected by incubating plants in 50 mM potassium phosphate pH 7 containing 0.1% (v/v) triton X-100, 1 mM potassium ferricyanide, 1 mM potassium ferrocyanide, 10 mM EDTA and 575 μM X-Gluc at 37°C for 16 h.

## Authors' contributions

MT generated the double mutants, carried out the morphological characterization of single/double mutants, hormone response assays and analysis of *AXR3::GUS *expression, contributed toward the mapping and design of the study, and drafted the manuscript. JJ performed the analysis of axr3 protein turnover, *ARF19 *allelism tests, and contributed toward the mapping and experimental design. PS carried out the mutant screen, isolated the *pax1-1 *single mutant and participated in the design of the study. DR generated the AXR3::GUS reporter line. OL participated in the design and coordination of the study and helped to draft the manuscript. All authors read and approved the final manuscript.

## Figures and Tables

**Table 1:** Timing of *pax1-1 *phase change

	Number of juvenile leaves	Timing of floral transition (dag)	Number of leaves at floral transition
Wild type (Columbia) n = 20	5.8 ± 0.09	16.50 ± 0.29	10.05 ± 0.20
*pax1-1* n = 10	8.60 ± 0.37	19.20 ± 0.44	12.50 ± 0.52

For each sample, the mean and SE are shown.
